# Bacteroidetes promotes esophageal squamous carcinoma invasion and metastasis through LPS-mediated TLR4/Myd88/NF-κB pathway and inflammatory changes

**DOI:** 10.1038/s41598-024-63774-6

**Published:** 2024-06-04

**Authors:** Zhongbing Wu, Jianxin Guo, Zhenhan Zhang, Shuang Gao, Ming Huang, Yu Wang, Yushuang Zhang, Qinghuan Li, Jing Li

**Affiliations:** 1 College of Integrated Chinese and Western Medicine, Hebei Medical University, Shijiazhuang, 050017 China; 2 The Fourth Hospital of Hebei Medical University, Shijiazhuang, 050011 China; 3 Department of Traditional Chinese Medicine, ShiJiaZhuang Medical College, Shijiazhuang, 050011 China

**Keywords:** 16S rRNA, Bacteroides, LPS, EMT, ESCC, Cancer, Metastasis, Bacterial toxins, Metagenomics

## Abstract

Gut microbiota plays a crucial role in gastrointestinal tumors. Additionally, gut microbes influence the progression of esophageal cancer. However, the major bacterial genera that affect the invasion and metastasis of esophageal cancer remain unknown, and the underlying mechanisms remain unclear. Here, we investigated the gut flora and metabolites of patients with esophageal squamous cell carcinoma and found abundant *Bacteroides* and increased secretion and entry of the surface antigen lipopolysaccharide (LPS) into the blood, causing inflammatory changes in the body. We confirmed these results in a mouse model of 4NQO-induced esophageal carcinoma in situ and further identified epithelial–mesenchymal transition (EMT) occurrence and TLR4/Myd88/NF-κB pathway activation in mouse esophageal tumors. Additionally, in vitro experiments revealed that LPS from *Bacteroides* fragile promoted esophageal cancer cell proliferation, migration, and invasion, and induced EMT by activating the TLR4/Myd88/NF-κB pathway. These results reveal that *Bacteroides* are closely associated with esophageal cancer progression through a higher inflammatory response level and signaling pathway activation that are both common to inflammation and tumors induced by LPS, providing a new biological target for esophageal cancer prevention or treatment.

## Introduction

The incidence of esophageal cancer has demonstrated significant differences, with the north-central region of China being the main high-incidence area of esophageal squamous cell carcinoma (ESCC)^1^. The incidence of ESCC is comparatively insidious and often progresses rapidly, with a high incidence of invasion and distant metastasis, resulting in high mortality rates^2^.

The mechanisms of cancer metastasis are complex, and studies have revealed that disturbances in the intestinal microbiota may increase the secretion of certain metabolites that activate inflammatory and tumor-related signaling pathways in the body, thereby promoting cancer invasion and metastasis^3^. Patients with gastrointestinal cancers frequently exhibit pathological characteristics of intestinal dysbiosis, and the intestinal microbiota and its metabolites induce long-term chronic inflammatory activation of metastasis-related pathways in tumors^4^. Thus, the gut microbiota contributes to the development and progression of many gastrointestinal malignancies by modulating the cancer-inflammatory microenvironment. Esophageal cancer is a prevalent malignant tumor of the gastrointestinal tract, and studies have revealed that the occurrence of esophageal adenocarcinoma is closely associated with intestinal microbiota dysbiosis^5^. However, changes in the intestinal flora and their regulatory mechanisms for the invasion and metastasis of ESCC remain unknown.

This study aims to investigate the characteristics and mechanisms of action of the intestinal flora in ESCC. This study revealed a significantly increased abundance of the gram-negative bacterium *Bacteroides* in patients with ESCC and the mouse models of ESCC. Additionally, the blood of patients and mouse model demonstrated significant alterations in the lipopolysaccharide (LPS) and the inflammatory cytokines interleukin (IL)-6, IL-10, tumor necrosis factor-alpha (TNF-α), and transforming growth factor beta (TGF-β). LPS is the main functioning component of the outer membrane of *Bacteroides*^6^, and an increased abundance of *Bacteroides* increases the amount of free bacterial component LPS that enters the circulation^7^, thereby changing the inflammatory environment of the organism and ultimately providing favorable conditions for ESCC invasion and metastasis. We further confirmed the role and possible mechanism of LPS, the surface antigen of *Bacteroides*, in promoting invasion, metastasis, and epithelial–mesenchymal transition (EMT) in ESCC at the cellular level. The results of this study may provide a new target for ESCC treatment.

## Result

### Basic participant characteristics

This study included 30 patients with ESCC and 30 healthy controls. Table 1 shows the demographic characteristics of all included individuals. No significant differences in terms of age, gender, alcohol consumption, or smoking were found between the two groups of participants (*p* > 0.05).

**Table 1:** Descriptive data of included subjects in the study.

Demographic characteristic	No. of patients in group:		*P* value
	Control (*n* = 30)	ESCC (*n* = 30)	
Age (year)			
< 50	1	2	0.8430
50–59	3	4	
60–69	18	19	
≥ 70	8	5	
Sex			
Female	14	9	0.1904
Male	16	21	
Smoking history			
Smoker	17	19	0.6055
Nonsmoker	13	11	
Drinking history			
Drinker	17	21	0.2918
Nondrinker	13	9	
Stage			
I		6	
II		13	
III		7	
IV		4	

### *Bacteroides* induce inflammatory changes via LPS in patients with ESCC

**Figure 1:**
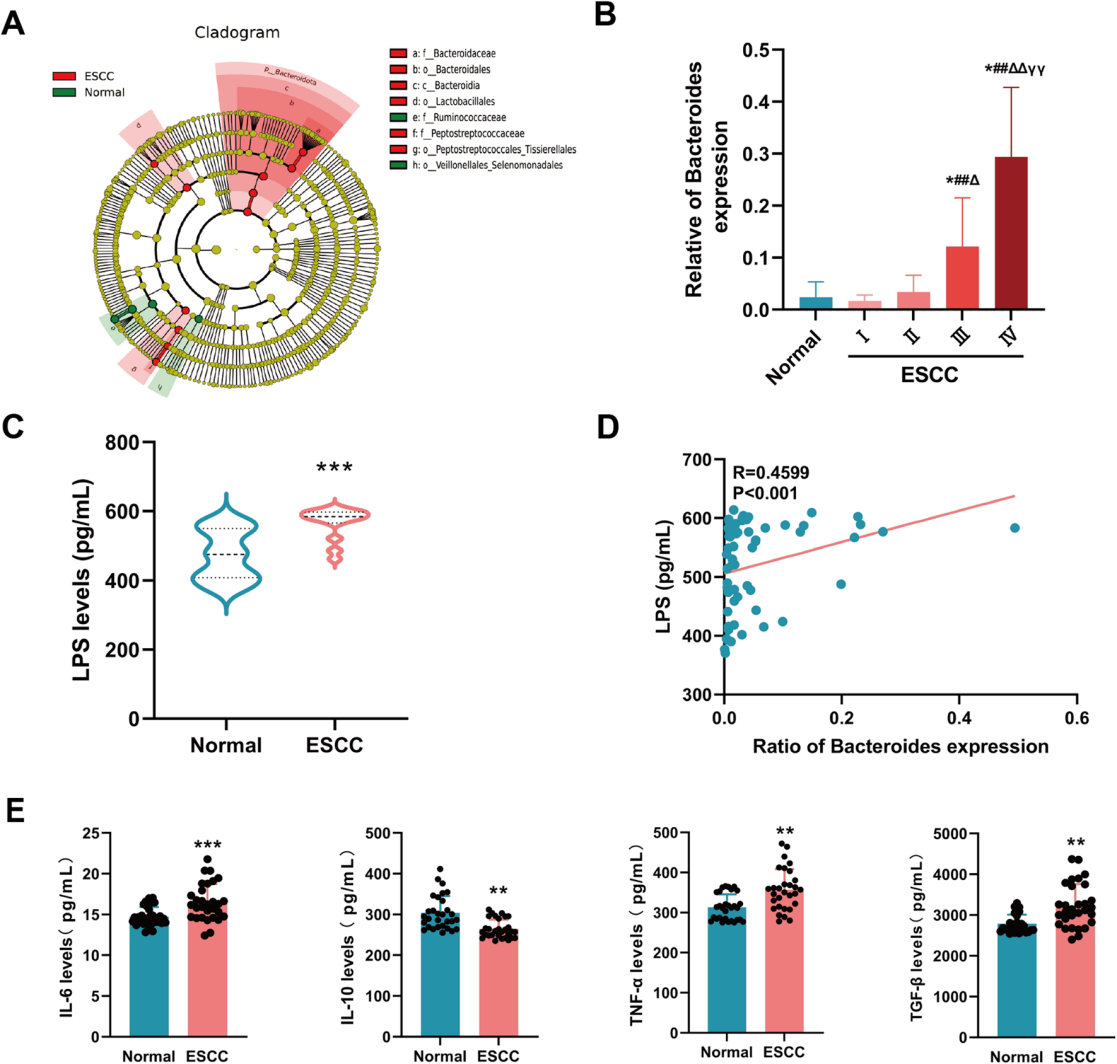
Expression of *Bacteroides*, LPS, and inflammatory factors in normal population and patients with esophageal cancer. (A) Evolutionary branching diagram showing species differentially enriched in the gut microbiota. Red indicates greater expression in ESCC than in controls, green denotes greater expression in controls than in ESCC, and yellow denotes no significant differences. (B) Comparison of the abundance of *Bacteroides*, *n* = 30. (C) Serum LPS levels, *n* = 30. (D) Correlation between *Bacteroides* and LPS levels with Spearman correlation. (E) Serum inflammatory cytokine levels, including IL-6, IL-10, TNF-α, and TGF-β, *n* = 30. * vs. controls, # vs. ESCC (I), Δ vs. ESCC (II), Y vs. ESCC (III), *p < 0.05, **p < 0.01, ***p < 0.001.

We revealed that the alphadiversity (species richness) of gut flora species in the two populations demonstrated a reasonable progressive amount of sample sequencing data, and the betadiversity (microbial composition) exhibited a large difference in gut microbial structure between the two populations (Fig. S1A–B). We used the LEfSe analysis of effect size method to determine differentially enriched species and identified Family *Bacteroidaceae*, Order *Bacteroidales*, Class *Bacteroidia*, and Order *Lactobacillales* in the fecal flora of patients with ESCC compared to healthy controls. Additionally, Family *Peptostreptococcaceaeg* and Order *Peptostreptococcales Tissierellaleh* increased in abundance, whereas Family *Ruminococcaceae* and Order *Veillonellales Selenomonadales* decreased in abundance, with a clear dominance of gram-negative *Bacteroides* (Fig. 1A). More specifically, we observed an increased abundance of *Bacteroides* with ESCC progression (Fig. 1B). Moreover, we detected LPS levels in the serum of clinical subjects using enzyme-linked immunosorbent assay (ELISA) and revealed significantly elevated LPS in the serum of patients with esophageal cancer (Fig. 1C). We further analyzed the data using Spearman’s correlation analysis to elucidate the relationship between *Bacteroides* and LPS and found that LPS was significantly and positively correlated with *Bacteroides* spp. (R = 0.4599, *P* < 0.001) (Fig. 1D).

Meanwhile, changes in the levels of endogenous bioactive cytokines, including IL-6, IL-10, TNF-α and TGF-β, which are usually diagnosed as indicators of cancer progression, were further assessed by ELISA of serum IL-6, IL-10, TNF-α, and TGF-β levels in clinical subjects, and we found significant differences in serum inflammatory cytokines in patients with ESCC compared with healthy controls (Fig. 1E).

### Inflammatory changes induced by *Bacteroides* via LPS in mouse ESCC models

**Figure 2:**
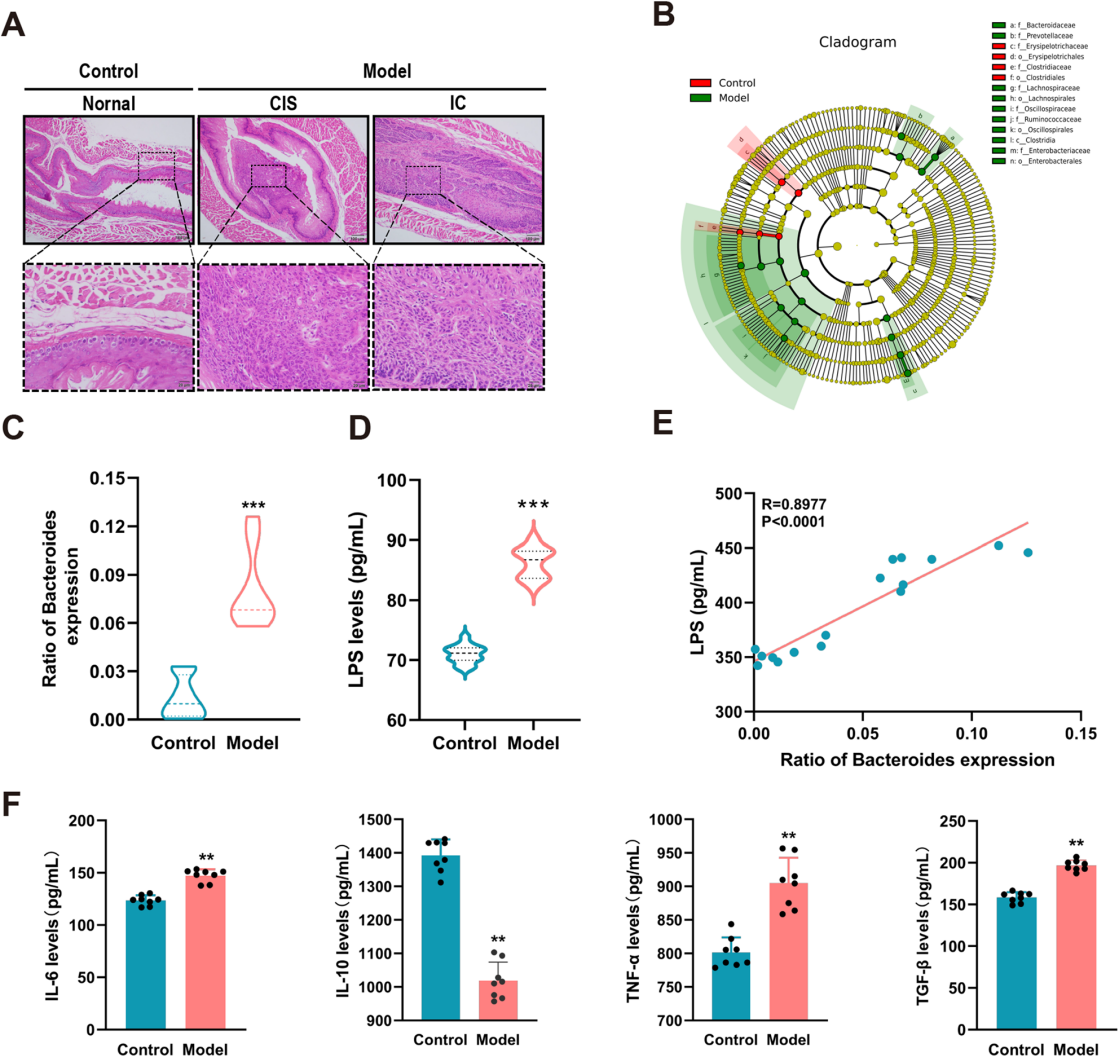
Expression of *Bacteroides*, LPS, and inflammatory factors in the experimental mouse. (A) Hematoxylin and eosin examination of histopathological changes. (B) Evolutionary branching diagram illustrating species differentially enriched in the gut microbiota. (C) Comparison of abundance of *Bacteroides*, *n* = 8. (D) Serum LPS levels, *n* = 8. (E) Spearman’s analysis method to analyze the correlation between *Bacteroides* and LPS levels in mice. (F) Inflammatory cytokines, including IL-6, IL-10, TNF-α, and TGF-β in mice, *n* = 8. **p < 0.01, ***p < 0.001.

We developed a 4NQO-inducible mouse model of ESCC to further investigate the association between fecal microorganisms and serum inflammatory factors. Figure 2A shows esophageal carcinoma in situ and invasive carcinoma in mice at week 32. The fecal flora of experimental mice was investigated using 16S rRNA technology. Alphadiversity (species richness) of mouse fecal flora species demonstrated a progressively reasonable amount of sequencing data from the samples, and betadiversity (microbial composition) exhibited large differences in the structure of gut microorganisms between the two groups of mice (Fig. S2A–B). Among them, Family *Bacteroidaceaeb*, Family *Prevotellaceaec*, Family *Lachnospiraceaeh*, Order *Lachnospiralesi*, Family *Oscillospiraceaej*, Family *Ruminococcaceaek*, Order *Oscillospiralesl*, Class *Clostridium*, Family *Enterobacteriaceae* with increased abundance of Order *Enterobacterales*, Family *Erysipelotrichaceaed*, Order *Erysipelotrichalese*, Family *Clostridiaceaef*, and Order *Clostridialesg* decreased in abundance. Interestingly, *Bacteroides* abundance was significantly higher in the resolving flora of the model mice than in the control group, which is similar to the findings of the clinical trial (Fig. 2B–C). Meanwhile, the serum LPS level of mice in the model group was significantly increased (Fig. 2D), and Spearman’s correlation analysis revealed that LPS was significantly and positively correlated with *Bacteroides* (R = 0.8977, *P* < 0.0001) (Fig. 2E).

Moreover, compared with the normal controls, significant changes in serum IL-6, IL-10, TNF-α, and TGF-β levels were observed in the ESCC mouse model and were consistent with the changes in inflammatory cytokines in patients with ESCC (Fig. 2F).

### Gut barrier damage increases circulating LPS

**Figure 3:**
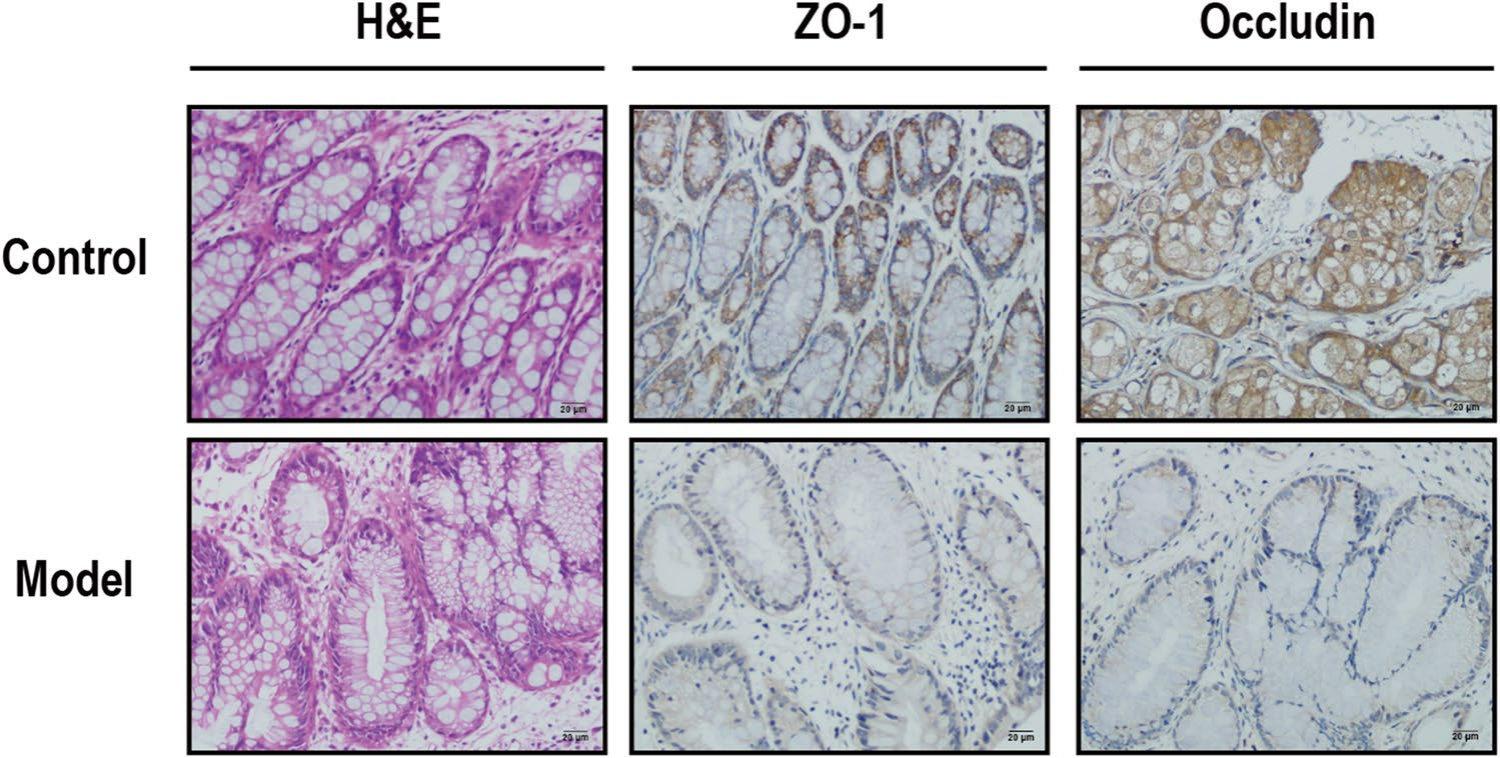
Disruption of the intestinal barrier and increased permeability in esophageal cancer mice. Histopathological changes in the rectum were examined by H&E under a microscope, H&E: hematoxylin and eosin. ZO1 and Occludin staining of mouse rectum tissue, brown for positive staining; ZO1 and Occludin: Mouse intestinal barrier-associated proteins. Observed under a 40 × microscope.

Immunohistochemistry (IHC) was used to detect mouse intestinal barrier-associated proteins, zonula occludens 1 (ZO-1) and occludin. The results revealed a significant decrease in ZO-1 and occludin-positive staining in the model group compared to that in the control group (Fig. 3). This indicates a damaged intestinal barrier in mice, increased permeability, and elevated LPS entry into the blood through the intestinal mucosal barrier.

### Increased tumor proliferation and EMT and activation of TLR4/Myd88/NF-κB signaling pathway in model mice

**Figure 4:**
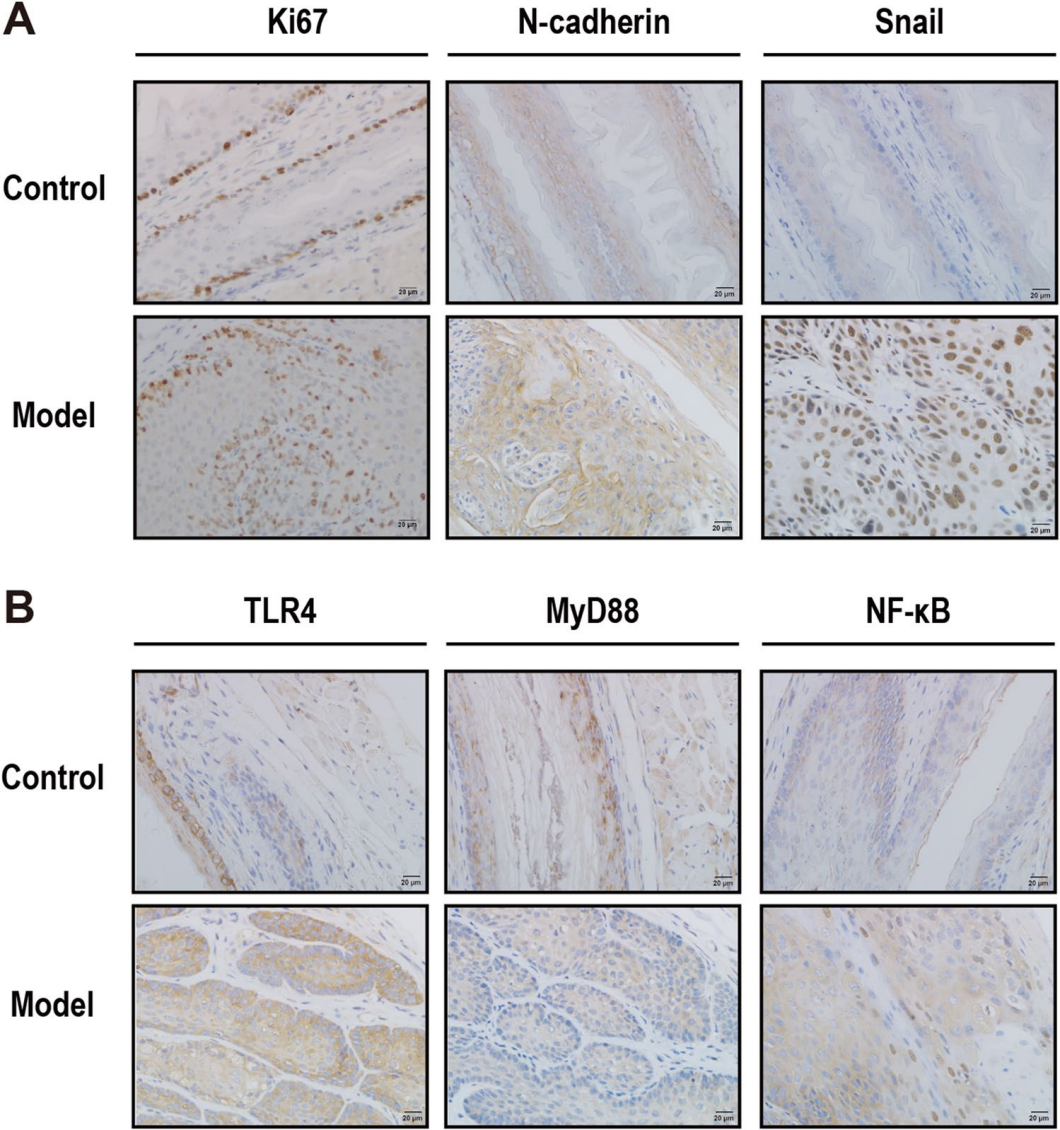
Expression of TLR4/Myd88/NF-κB signaling pathway and EMT-related proteins in ESCC tissues. (A) Immunohistochemistry (IHC) staining of Ki67, E-cadherin, and snail in mouse esophageal tissues at 40 × magnification. (B) IHC staining of TLR4, MyD88, and NF-κB in mouse esophageal tissues at 40 × magnification.

IHC revealed that the proliferation marker protein Ki67, invasion and metastasis marker proteins N-cadherin, and snail of ESCC tumor tissue were significantly increased in the model group (Fig. 4A). Additionally, IHC detected the expression and distribution of TLR4, Myd88, and NF-κB. The three indicators demonstrated increased expression in esophageal epithelial tissue compared to that in the control group (Fig. 4B). This indicates that EMT progression in mouse esophageal cancer may be associated with the TLR4/Myd88/NF-κB signaling pathway.

### LPS promotes KYSE-150 cell proliferation and migration invasion

**Figure 5:**
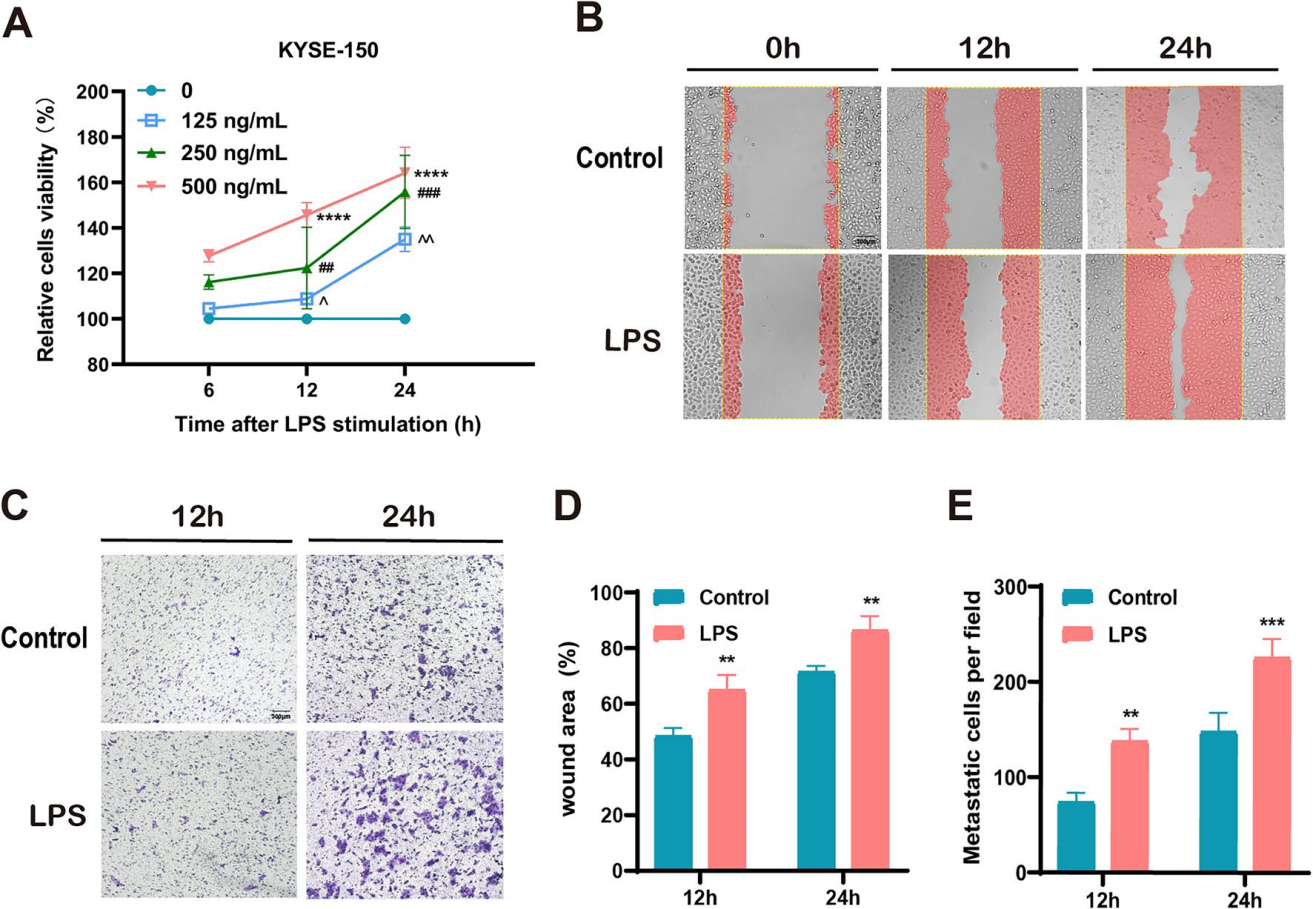
LPS promotes KYSE-150 cell proliferation, migration, and invasion. (A) Cell viability at different LPS concentrations (0, 125, 250, and 500 ng/mL) at 6,12, and 24 h. (B) Cell migration ability with LPS at 500 ng/ml. (C) Cell invasion ability of LPS at 500 ng/ml for 12 or 24 h. **p < 0.01,***p < 0.001,****p < 0.0001.

Studies have revealed that *Pseudomonas fragilis* is the most pathogenic of the genus *Pseudomonas*^8^. We obtained LPS for in vitro experiments by culturing *P. fragilis*. We used different LPS concentrations (0, 125, 250, and 500 ng/mL) to coculture with KYSE-150 cells for 6, 12, and 24 h. The proliferative ability of KYSE-150 cells was significantly improved after LPS treatment dose- and time-dependent manner (Fig. 5A). Additionally, LPS of 500 ng/mL was used to treat KYSE-150 cells for 12 and 24 h to observe their migration and invasion abilities. The results revealed significantly improved migration and invasion abilities of KYSE-150 cells in the LPS intervention group compared with the control group (Fig. 5B–E).

### LPS promotes EMT in KYSE-150 cells and activates the TLR4/Myd88/NF-κB pathway

**Figure 6:**
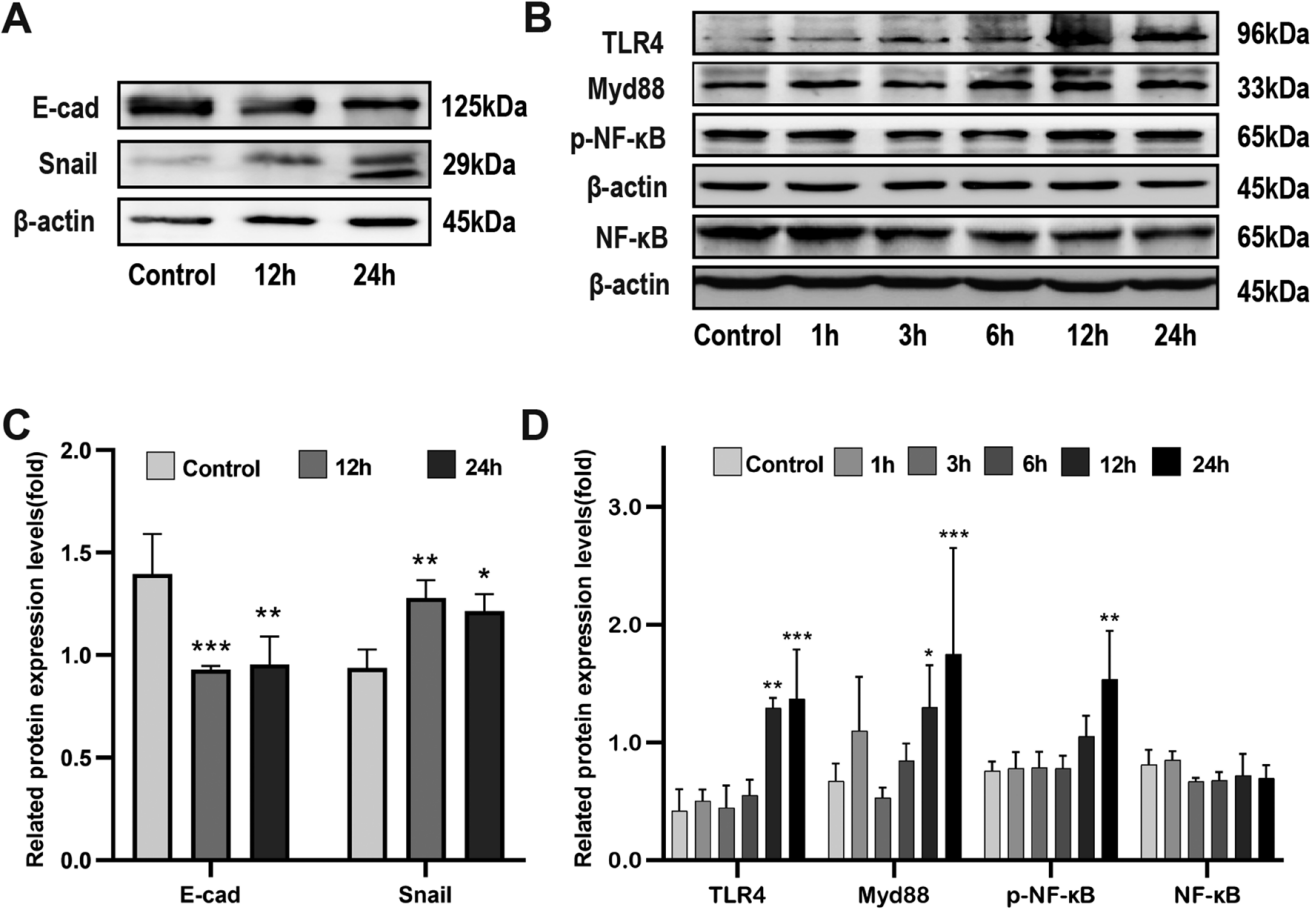
LPS promotes EMT activation of TLR4/Myd88/NF-κB pathway in KYSE-150 cells. (A, C) E-cadherin and snail expression after LPS treatment. (B, D) TLR4, myd88, p-NF-κBp65, and NF-κBp65 protein expression after LPS treatment, *n* = 3. *p < 0.05, **p < 0.01, ***p < 0.001. Original blots are presented in plementary Figs 3, 4, 5, 6, 7 and 8.

We investigated the expression of key EMT proteins in LPS-treated KYSE-150 cells and found that the epithelial marker E-cadherin was significantly downregulated, and the mesenchymal markers N-cadherin and snail were significantly upregulated at both 12 and 24 h (Fig. 6A, C).

We explored the molecular mechanisms by which LPS promoted KYSE-150 cell proliferation, migration, and invasion. We examined the expression of TLR4/Myd88/NF-κB pathway-related proteins after LPS treatment of KYSE-150 cells. Western blot analysis revealed significantly elevated TLR4 and myd88 at 12 versus 24 h, and p-NF-κBp65 was significantly increased at 24 h. However, NF-κBp65 exhibited no significant changes. This indicated that the TLR4/Myd88/NF-κB pathway was activated by LPS in the order of signal transduction (Fig. 6B, D).

## Discussion

Gut microbiota plays an important role in cancer development and is frequently characterized by altered microbiota in cancer. Gut microbiota alterations play an important role not only in colorectal cancer^9^ but also in extraintestinal tumors, including melanoma^10^, hepatocellular carcinoma^11^, and gastric cancer^12^. A related study on esophageal cancer by Natasha et al. revealed that a high-fat diet accelerates esophageal allopatric hyperplasia by altering the esophageal microenvironment and gut microbiome^5^. Additionally, Qunate et al. revealed that metabolites of intestinal microorganisms are important mediators in accelerating the progression of esophageal cancer^13^.

Previous studies revealed the involvement of microbiota changes in the progression of ESCC in patients with esophagitis and ESCC^14^. We performed microbiota analysis of fresh feces from patients with ESCC using the 16S rRNA technique to investigate the critical role of major strain changes and their metabolites in ESCC development, and the results revealed a significantly elevated abundance of gram-negative bacteria, *Bacteroides*, in all cases. Interestingly, both gastric and colorectal cancers demonstrated a significant increase in the abundance of *Bacteroides*^15,16^. Our step studies revealed a positive correlation between serum LPS and *Bacteroides* abundance in patients with ESCC and the ESCC mouse model.

Meanwhile, experiments revealed an increased inflammatory infiltration level in the rectal tissues of mice in the model group and decreased expression of tight junction proteins, occluding, and ZO-1. Occludin and ZO-1 are the most abundant components of intercellular tight junction formation^17^. This indicates intestinal barrier damage and increased intestinal permeability in mice^18,19^. LPS can not only damage the intestinal tract but also trigger bacterial translocation, impair the intestinal barrier, and lead to abnormal expression of intestinal tight junction proteins. This allows LPS to penetrate the intestinal wall^20^. The increase in LPS in the blood further activates the host immune response^21^, dysregulating the cancer-inflammatory microenvironment, which plays an important role in extraintestinal diseases^22,23^. Therefore, we hypothesized that the increased abundance of *Bacteroides* in the intestinal flora plays an important role in ESCC development and that its surface antigen-LPS may be a key factor.

Our study revealed that elevated serum IL-6, TNF-α, and TGF-β and decreased IL-10 levels revealed that patients with ESCC and model mice demonstrated increased immune levels, and these inflammatory cytokines are considered important mediators associated with inflammation and cancer. Imbalances in pro- and antiinflammatory signaling factors promoted inflammatory cancer transformation and cancer progression^24^. IL-6 is considered a pleiotropic molecule that promotes tumor transformation and can, particularly, promote tumor cell invasion and metastasis^25^. IL-10 is involved in suppressing inflammatory responses, promoting tissue repair, and maintaining immune tolerance^26^. Under normal conditions, IL-10 is involved in maintaining immune tolerance in the gut microbiota^27^, which limits intestinal inflammation and tumor formation^28^. Many studies revealed that the effects of TNF-α and TGF-β include the promotion of EMT invasion and metastasis^29–32^.

Research revealed that LPS generated a broad inflammatory cascade response by binding to TLR4^33^. TLR4 is not only a key hub that connects innate and adaptive immunity but also a sensor for recognizing microbial ecological dysregulation, which reflects the dysregulation of the body’s immunity as well as the microbiota and is closely related to tumors^34,35^. Sato et al. revealed that high TLR4 expression predicts poor prognosis in patients with advanced thoracic ESCC after esophagectomy^36^. Upon LPS stimulation, TLR4 uses MyD88 junction protein to transmit signals that activate NF-κB and induce inflammatory cytokines^37,38^. Recent studies revealed that TLR4/Myd88/NF-κB is a signaling pathway that improves IL-6, TNF-α, and TGF-β production^39–41^. The results of this study indicate significantly increased protein expression of key nodes of the TLR4/Myd88/NF-κB pathway in the esophagus of mice modeled for esophageal cancer in situ, and the same changes in protein expression were observed in esophageal cancer cells after LPS treatment in vitro. This indicates that the TLR4/Myd88/NF-κB signaling pathway is involved in esophageal cancer disease progression and is closely associated with LPS.

EMT is abnormally activated in human cancers, providing a special capacity for tumor cell movement that contributes to tumor cell invasion^42^. Some evidence indicates that the TLR4/Myd88/NF-κB signaling pathway, as well as inflammatory cytokines, influenced the EMT process^43–46^. We performed in vivo and in vitro experiments to further validate the effect of LPS on the EMT process in esophageal cancer and revealed the accelerated EMT process progression in ESCC, which provides invasive and migratory properties to cancer cells.

This study has several limitations. The human gut microbiota is a diverse ecosystem that is closely related to cancer; however, we only investigated the promotional effects and possible mechanisms of *Bacteroides*, which were the most significant differences in our study. And the roles of gut microorganisms in ESCC still need to be collected from more clinical samples and experimental data and investigated in depth. Additionally, due to ethical issues, we were unable to obtain intestinal tissue samples from esophageal cancer patients and normal volunteers to compare the extent of intestinal barrier disruption between the two groups.

## Conclusions

**Figure 7:**
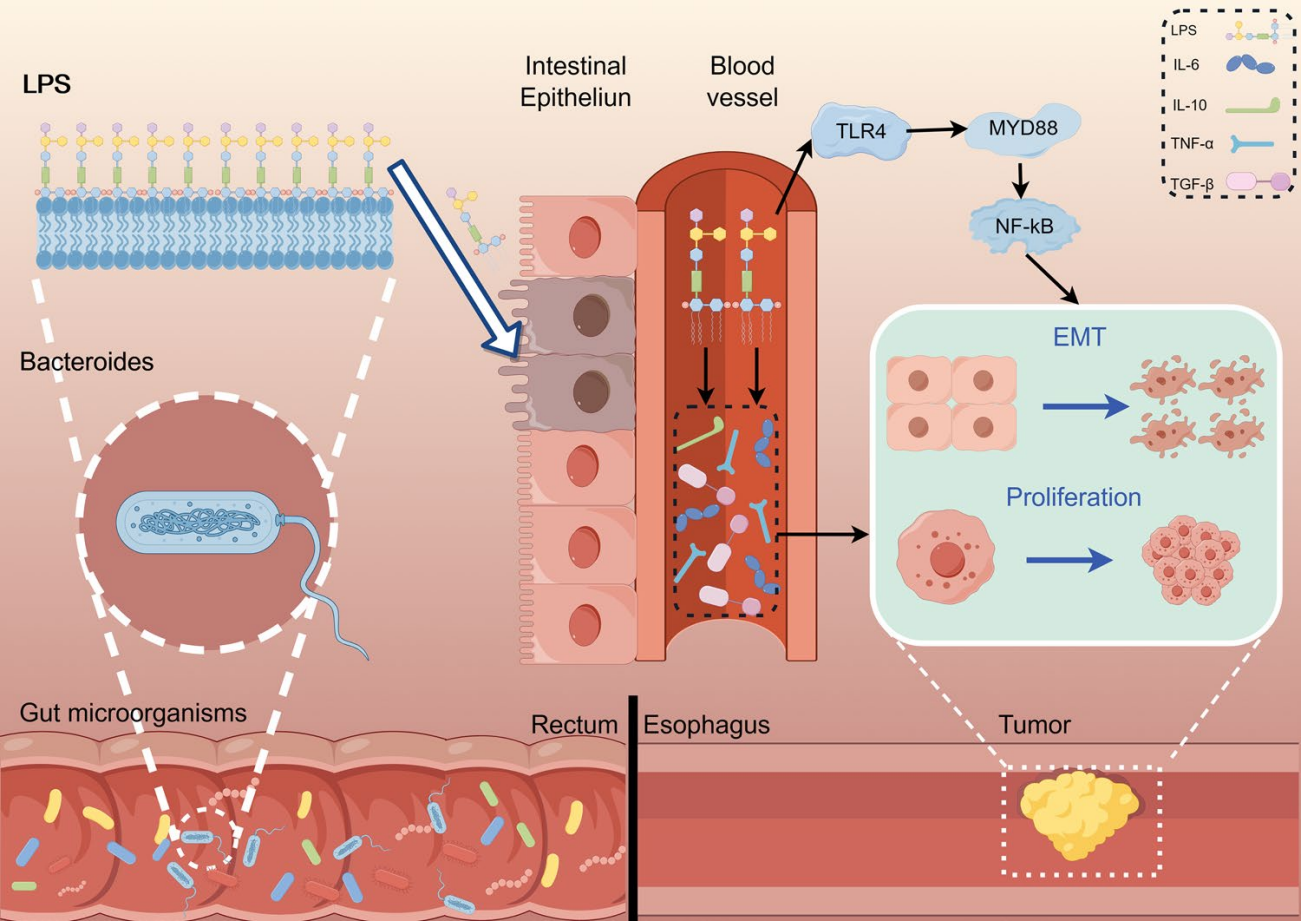
Schematic representation of the mechanism by which Bacteroidetes promotes migratory invasion of esophageal cancer cells.

Overall, the increased abundance of *Bacteroidetes* was associated with ESCC development. Additionally, *Bacteroidetes* may further initiate the EMT process through LPS-mediated alteration of the inflammatory environment and TLR4/Myd88/NF-κB signaling pathway, thereby promoting the invasion and metastasis of ESCC (Fig. 7).

## Materials and methods

### Clinical samples and ethical statement

This study included 30 patients newly diagnosed with esophageal cancer (ESCC group) and 30 healthy controls from the Department of Physical Examination (control group) of the Fourth Hospital of Hebei Medical University in 2021. Inclusion criteria^47^ were as follows: (1) patients aged > 18 years, (2) patients with primary esophageal cancer, (3) patients who had not received radiotherapy, chemotherapy, and/or surgery, and (4) all healthy controls with normal bowel habits. Exclusion criteria^48^ were as follows: (1) patients with diabetes and depression, (2) patients who had taken antibiotics, H2 receptor antagonists, proton pump inhibitors, and probiotics within the past 1 month, (3) patients with other cancers, and 4) dental bacterial diseases. The study was conducted in strict accordance with the protocol approved by the Ethics Committee of the Fourth Hospital of Hebei Medical University, and all subjects provided written informed consent (2022KY057). The ESCC group referred to the “Chinese Society of Clinical Oncology (CSCO) Esophageal Cancer Diagnosis and Treatment Guidelines-2020” developed by the CSCO, and diagnosed squamous cell carcinoma of the esophagus by histopathology.

### Animal models and experimental design

Female C57BL/6 mice (6 weeks old) were purchased from Beijing Wei Tong Li Hua Laboratory Animal Technology (License number: SCXK [Beijing] 2016–0011). The mice were housed in the Experimental Animal Center of the Fourth Hospital of Hebei Medical University (SPF level) following national and international guidelines. The Institutional Laboratory Animal Care Guidelines of the Fourth Hospital of Hebei Medical University approved the study (IACUC-4th Hos Hebmu-2022,003).

Freshly prepared 4NQO (N8141, Sigma-Aldrich) stock solution (in propylene glycol) was added to drinking water at 100 µg/mL, and the water was changed weekly. Mice were randomly categorized into an experimental group provided with drinking water containing 4NQO (*n* = 8) and a control group provided with drinking water without 4NQO (*n* = 8), but with the same volume of propylene glycol. Mice were allowed access to drinking water at all times during treatment. The mice were provided regular water after 16 weeks of 4NQO treatment^49,50^. Mice fasted for 12 h at the end of the experiment, were anesthetized with an intraperitoneal injection of sodium pentobarbital (100 mg/kg), and then sacrificed by cervical dislocation.

### Sample collection and 16S rRNA sequencing and analysis

Fresh fecal specimens were collected from the participants in sterile tubes, flash frozen in liquid nitrogen, and stored at − 80℃ refrigerator. Professional nurses performed intravenous blood collection in strict accordance with aseptic standard procedures. Serum was collected by centrifugation and stored at − 80 °C.

Samples were extracted from genomic DNA and amplified by polymerase chain reaction (PCR) applying primers specific to the 16SV34 region (upstream primer CCTAYGGGRBGCASCAG and downstream primer GGACTACNNGGGGTATCTAAT). The purified and gel electrophoresis-detected PCR products were recovered using a gel recovery kit (TIANGEN Biotech Co., Ltd., Beijing, China). The Illumina company library building kit (model: TruSeq DNA PCR Free Library Preparation Kit) was used to construct the library. NovaSeq6000 was used for computer sequencing, after qualifying the library for quantitative inspection. Changes in the abundance of bacteria were analyzed, and bacteria with large differences were selected as candidates.

### ELISA assay

ELISA kits (Shanghai Fusheng Industrial Co., Ltd., China) were used for detecting LPS, IL-6, IL-10, TNF-α, and TGF-β levels. Briefly, sera from clinical participants and experimental mice were processed to obtain a suitable supernatant, which was later processed following the ELISA kit instructions, and absorbance values (OD) of the samples were measured at 450 nm to quantify the LPS, IL-6, IL-10, TNF-α, and TGF-β values by comparing the values relative to each standard curve.

### Histochemical and immunohistochemical (IHC) staining

The esophageal and rectal tissues of mice were fixed with 4% paraformaldehyde, dehydrated, and paraffin-embedded at the end of the experiments to prepare 4-μm-thick sections, which were deparaffinized, hydrated, and other steps, and then subjected to hematoxylin and eosin staining with IHC staining.

The results in ESCC tissue included TLR4 (GB15186, Servicebio, China), Myd88 (GB11269, Servicebio, China), NF-κB (GB11269, Servicebio, China), Ki-67 (GB111141, Servicebio, China), E-cadherin (GB12083, Servicebio, China), and snail (GB11260, Servicebio, China) proteins, and rectal tissue used ZO1 (GB111402, Servicebio, China) and occludin (GB111401, Servicebio, China) proteins for IHC staining. Finally, two observers, who were unaware of each other, performed histologic evaluation and investigated the sections.

### Preparation of dried powder of LPS from *Mycobacterium fragilis.*

The powder of Pseudomonas fragilis (BNCC336948, BeNa Culture Collection, China) preserved in the ampoule tube was passaged in Columbia blood agar plates (BNCC352241, BeNa Culture Collection, China), and the plates were incubated in an anaerobic gas-producing bag (Mitsubishi motors, MGC C-01, Japan) in an anaerobic jar (Mitsubishi motors, MGC C-31, Japan) for 24–48 h at 37℃ for two consecutive passages (motors, MGC C-01, Japan) in an anaerobic tank (Mitsubishi motors, MGC C-31, Japan) for 24 h. Single colonies were selected and passaged into liquid thioglycolate medium FT tubes (BNCC353538, BeNa Culture Collection, China). Collection, Chin) and cultured in a 37℃ constant temperature incubator (ZHICHENG, ZXDP-B2080, China) for 24 h. The liquid medium was centrifuged at 8000 g for 3 min at room temperature (22 °C) to harvest the *B. fragilis* precipitate, and LPS samples were extracted from the bacterial precipitates using an LPS extraction kit (EX1740, Servicebio, China), and finally desalted and lyophilized using 10-KD ultrafiltration tubes, and stored at − 80 °C in a refrigerator.

### Cell culture

The human esophageal squamous cell carcinoma (ESCC) cell line KYSE150 was obtained from the Cell Bank of the Chinese Academy of Sciences (Shanghai, China). KYSE150 Cells were cultured in RPMI-1640 medium (Gibco, USA) containing 10% fetal bovine serum (Gibco, USA). Cells were cultured in a 37℃, 5% CO2 humidified incubator (Thermo, US).

### Cell counting kit-8 (CCK-8) Assay

Cells were plated in 96-well plates at 2 × 103 cells/well. Cells were incubated in media with various LPS concentrations (0, 125, 250, and 500 ng/ml, Sigma, Germany) for 6, 12, and 24 h. The CCK-8 solution was added at a 1:10 dilution into each well. A microplate reader (Bio-Rad Laboratories, Hercules, USA) was used to measure absorbance at 450 nm. Additionally, the results were expressed as the percentage of CCK-8 conversion relative to control cell absorbance.

### Scratch assay

The cells were inoculated in a 24-well culture plate overnight. We made straight scratches in middle of the well using 200-μl tips and gently washed three times with PBS to ensure get rid of floating cells. Cells were incubated in a medium containing 200 ng/mL LPS. Images were taken at 12 h and 24 h, and images were analyzed using Image J.

### Invasion assays

Transwell system based with Matrigel, which was produced from BD, USA, was used to evaluate the invasiveness of tumor cells. Control cells or LPS treated cells were added to the upper chamber, and bovine serum-containing medium was added to the lower chamber. In addition, these cells were cultured in a CO 2 incubator for 12 h and 24 h. Cells in the upper chamber were removed, and those passing through the chamber were fixed with 4% paraformaldehyde, and stained with crystal violet, and then the photographs were taken.

### Western blotting

WB analysis was conducted to evaluate protein expression levels. Cells were lysed using radioimmunoprecipitation assay buffer (G2002, Servicebio, China) on ice for 10 min. The enhanced bicinchoninic acid Protein Assay Kit (PC0020, Solarbio, China) was used to determine the quality of the total protein collected. The samples were then loaded onto an 8% SDS-PAGE gel and transferred onto a polyvinylidene fluoride membrane. Membranes were blocked with 5% bovine serum albumin in phosphate-buffered saline for 60 min. Subsequently, the membranes were horizontally cut following the molecular weight of the protein of interest, followed by incubation with primary antibodies against TLR4 rabbit pAb (1:2500, A5258, ABclonal), Myd88 rabbit pAb (1:250, A0980, ABclonal), P-NF-κB p105-S932 rabbit mAb (1:2500, AP1355, ABclonal), NF-κB rabbit pAb (1:250, A11160, ABclonal, China), snail rabbit pAb (1:750, A5243, ABclonal), E-cadherin rabbit pAb (1:1000, A3044, ABclonal), β-Actin rabbit mAb (1:50,000, AC026, ABclonal), and goat antirabbit IgG (H + L) as secondary antibody (1:5000, S1002, Ruipate) overnight at 4℃. The ECL detection kit (HY-K1005, MCE, USA) was used to detect protein expression, and ImageJ software was used for the analyses.

### Statistical analysis

All statistical data were analyzed with SPSS 23.0 software. Data were expressed as mean ± standard deviation. Student T-test was used to analyze the statistical difference; *p* < 0.05 indicated that the difference was statistically significant.

### Ethical approval

The research was conducted in strict accordance with the protocol approved by the Ethics Committee of the Fourth Hospital of Hebei Medical University, and written informed consent was obtained from all subjects (2022KY057). All procedures performed in studies involving human participants were in accordance with the ethical standards of the institutional and/or national research committee and with the 1964 Helsinki declaration and its later amendments or comparable ethical standards. The research was approved by the Institutional Laboratory Animal Care guidelines of Fourth Hospital of Hebei Medical University (IACUC-4th Hos Hebmu- 2022,003). This study was conducted appropriately and in accordance with local laws and institutional requirements. The experimental protocol was performed in accordance with the relevant guidelines and regulations of the Basel Declaration. The study is reported in accordance with ARRIVE guidelines (https://arriveguidelines.org).

### Supplementary Information

Supplementary Figures.

## Data Availability

Analysis of alpha/beta diversity of gut flora between the two populations is shown in Supplementary Fig. S1. Analysis of alpha/beta diversity of gut flora between the two groups of mice in Supplementary Fig. S1. All raw reads are stored in the NCBI Sequence Read Archive (SRA) database under accession number PRJNA1091832 (https://www.ncbi.nlm.nih.gov/bioproject/PRJNA1091832) and PRJNA1091827 (https://www.ncbi.nlm.nih.gov/bioproject/PRJNA1091827) and were available upon reasonable request to the corresponding authors. All the original Western blots with replicates of the experiments are available in Supplementary Figs 3, 4, 5, 6, 7 and 8.
