# Supplementary material for: Bacteroidetes promotes esophageal squamous carcinoma invasion and metastasis through LPS-mediated TLR4/Myd88/NF-κB pathway and inflammatory changes
Source: Sci Rep. 2024 Jun 4;14:12827. doi: 10.1038/s41598-024-63774-6 (PMC11150411; doi:10.1038/s41598-024-63774-6)
Supplement: Supplementary file 1 — Supplementary Figures. [file 41598_2024_63774_MOESM1_ESM.pdf]

Supplementary material

Supplementary Figure 1.

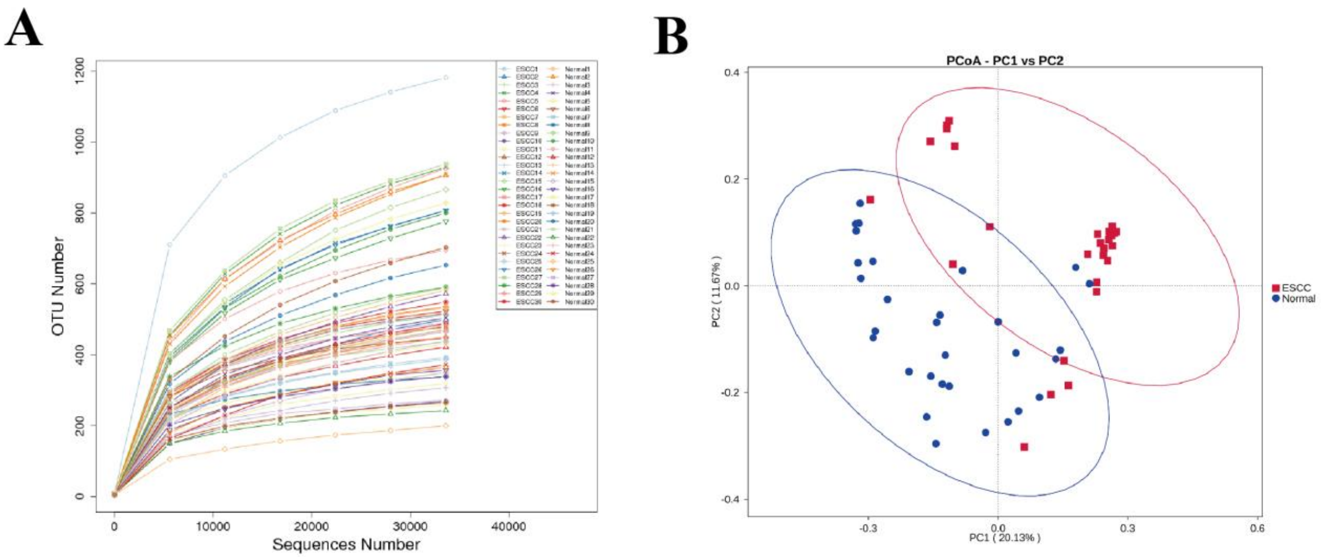

Supplementary Figure 2.

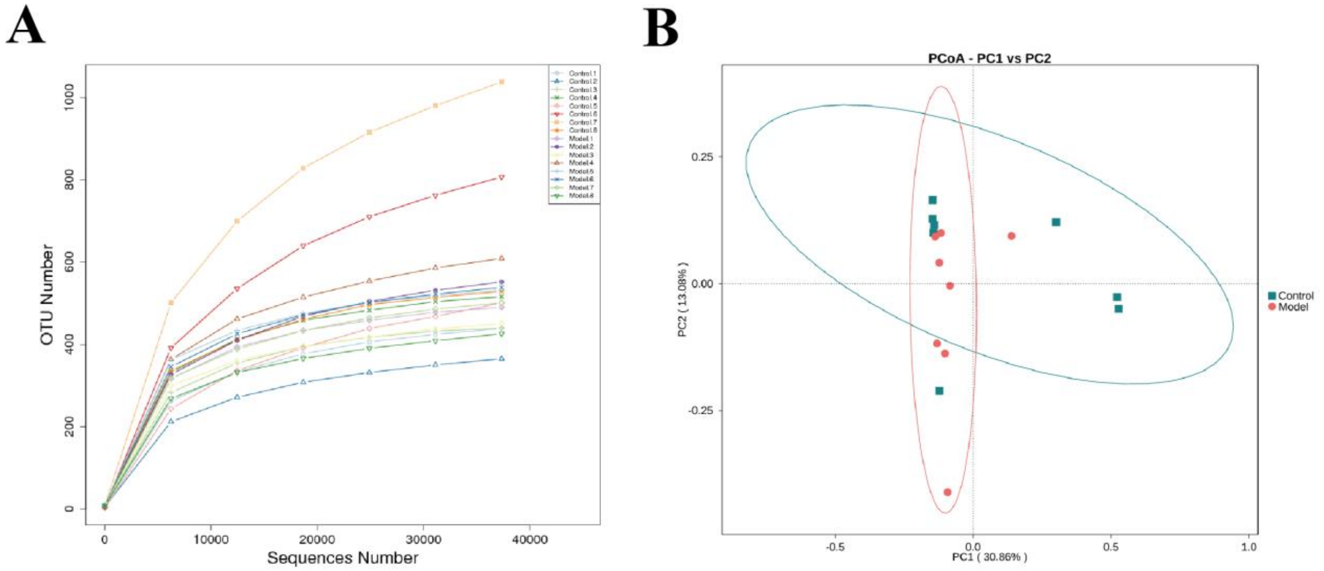

Supplementary Figure 3.

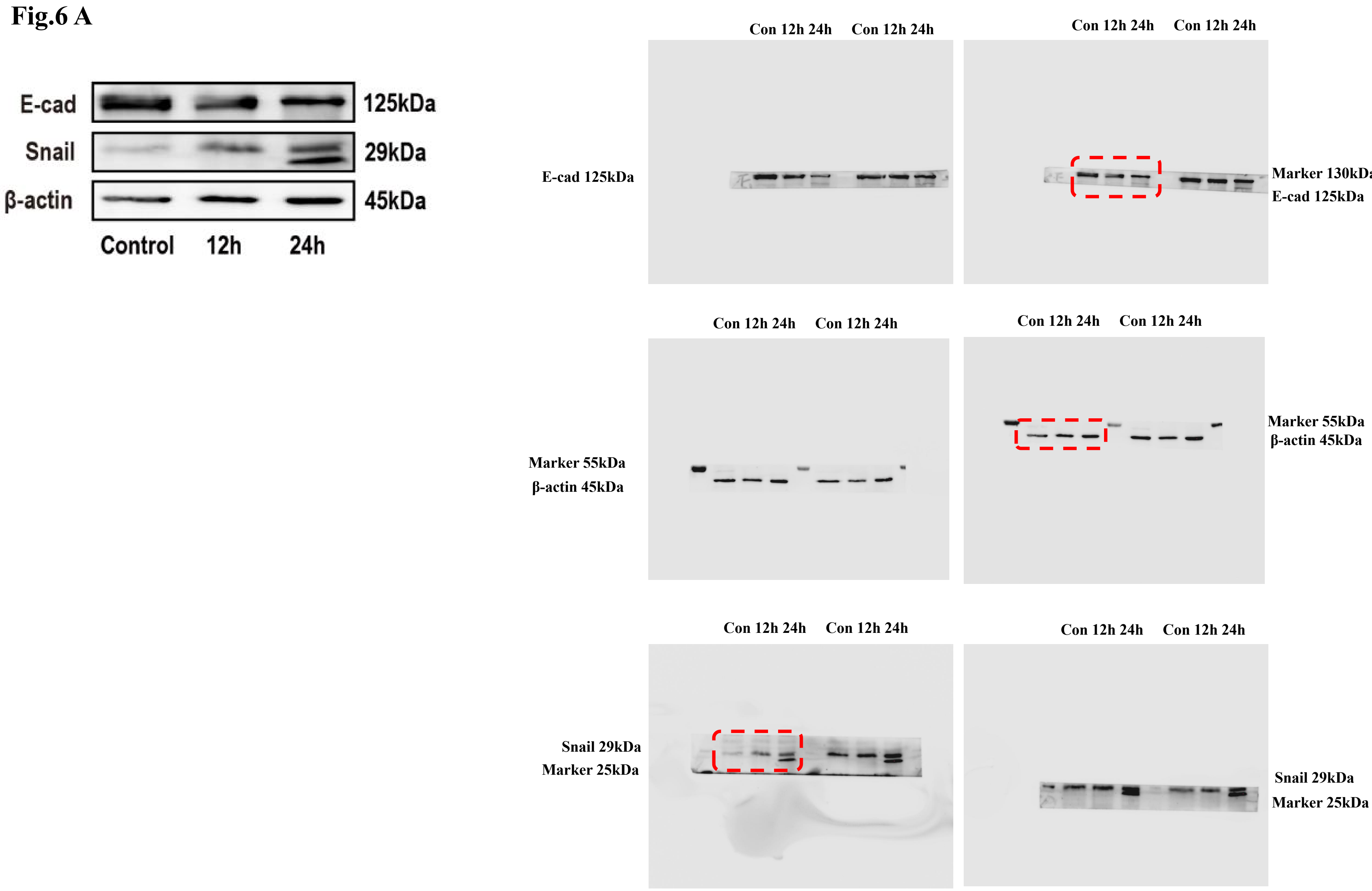

The red checkboxes are the bands presented in the manuscript image

Supplementary Figure 4.

Fig.6 B

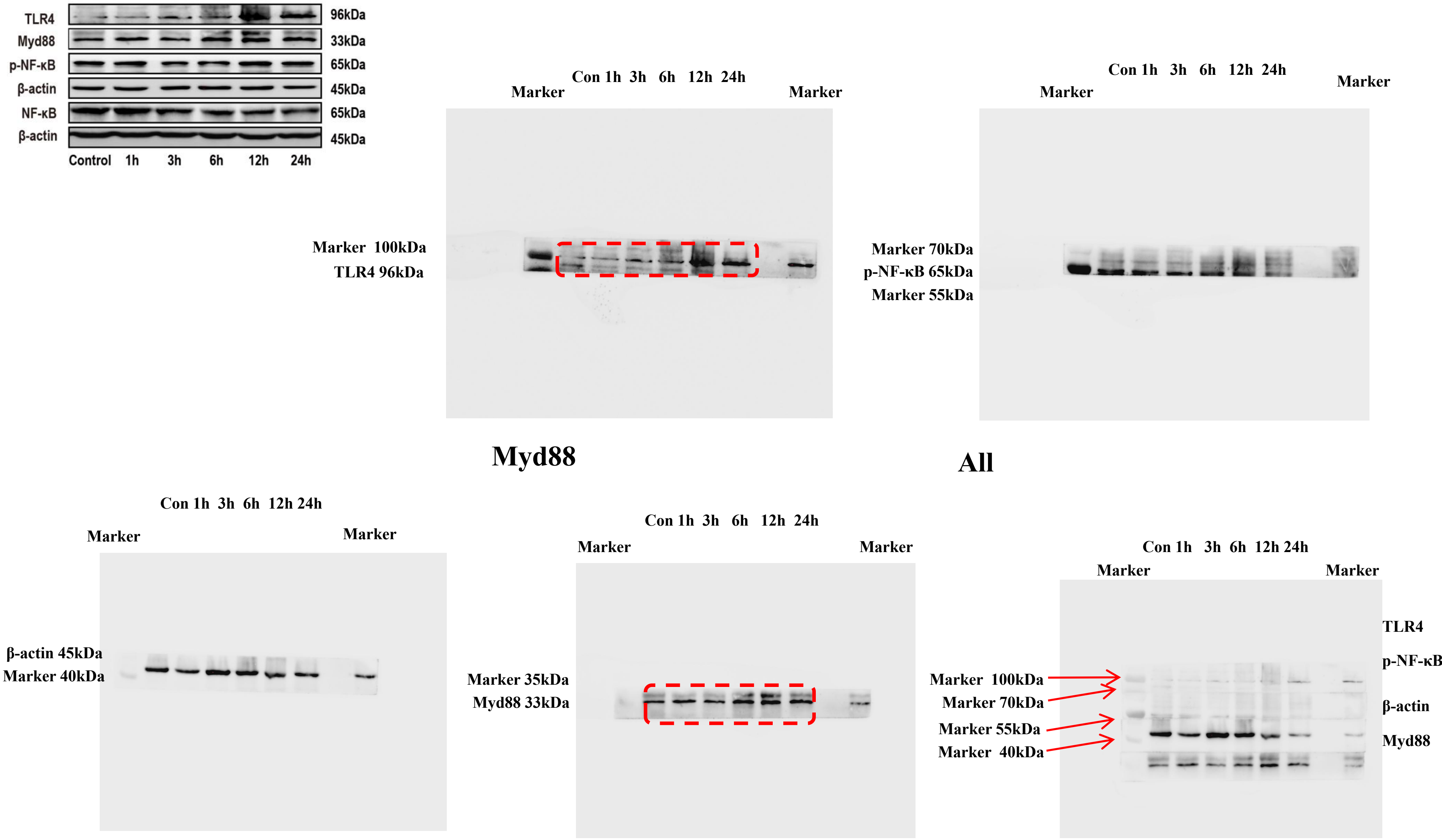

The red checkboxes are the bands presented in the manuscript image

Supplementary Figure 5.

Fig.6 B

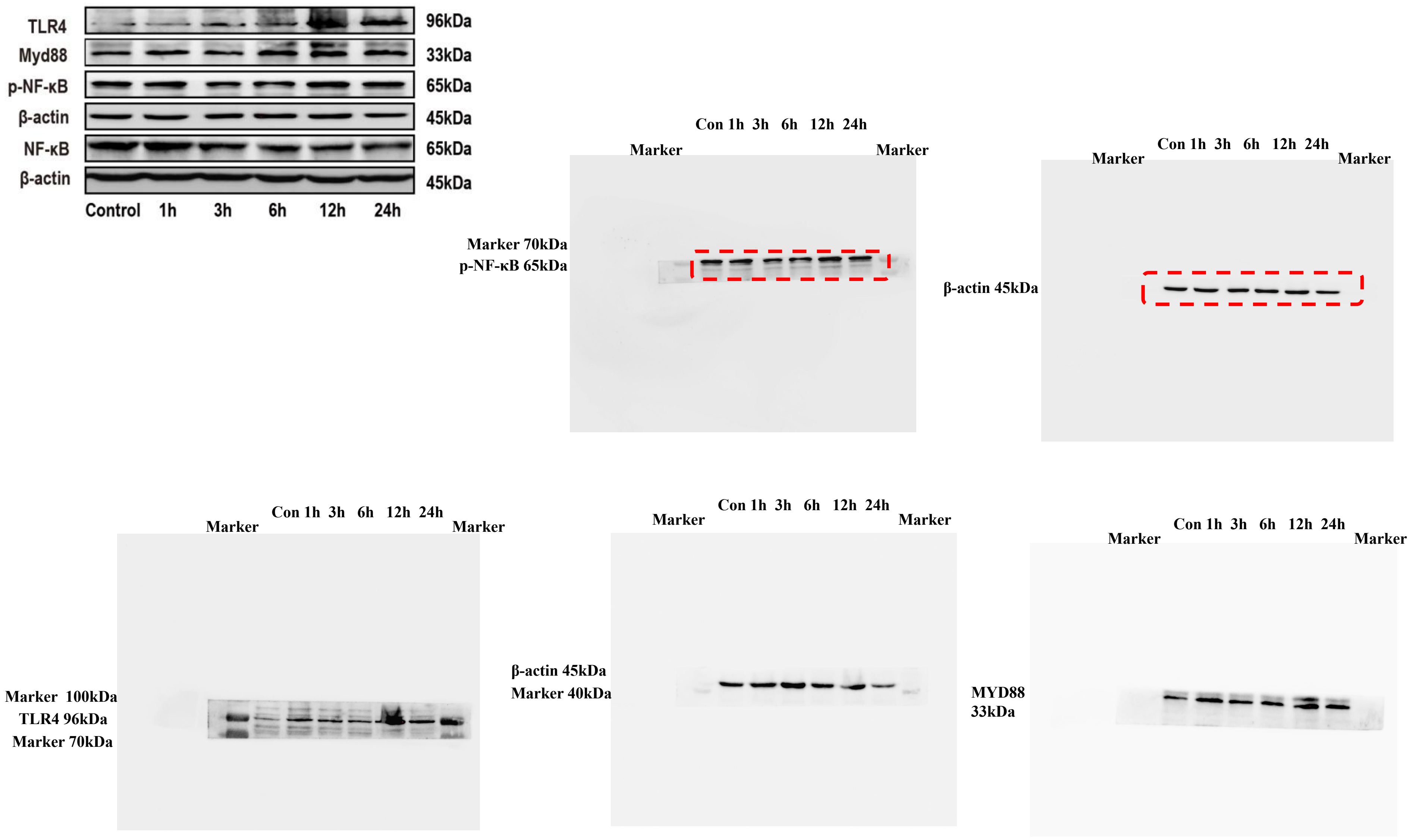

The red checkboxes are the bands presented in the manuscript image

Supplementary Figure 6.

Fig.6 B

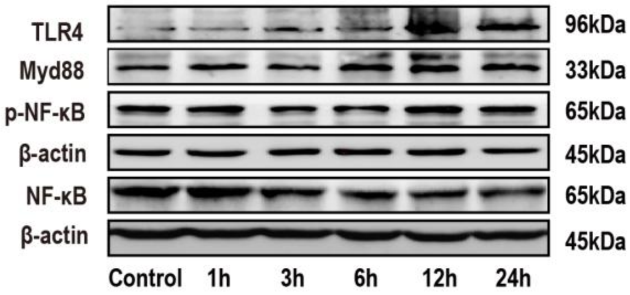

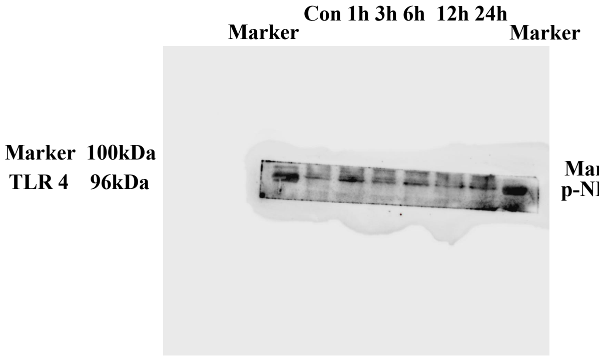

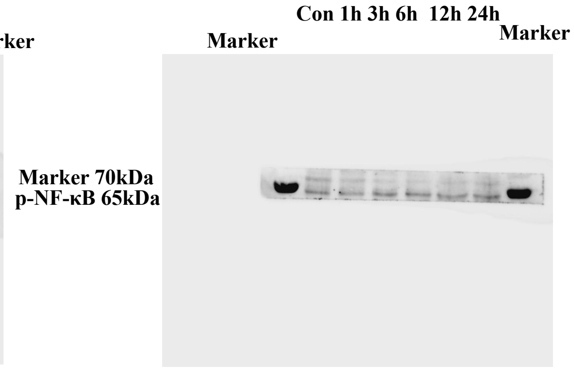

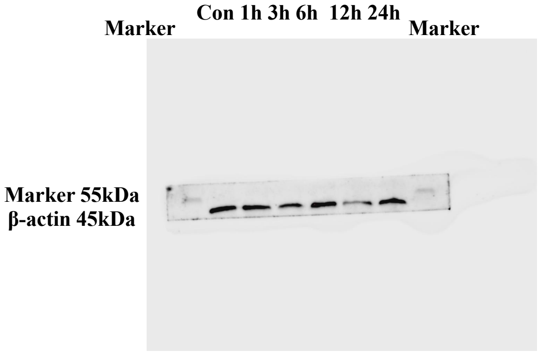

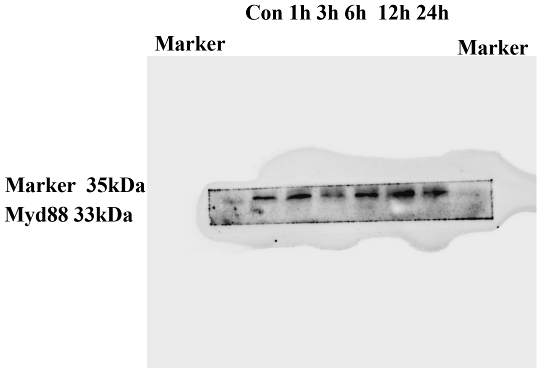

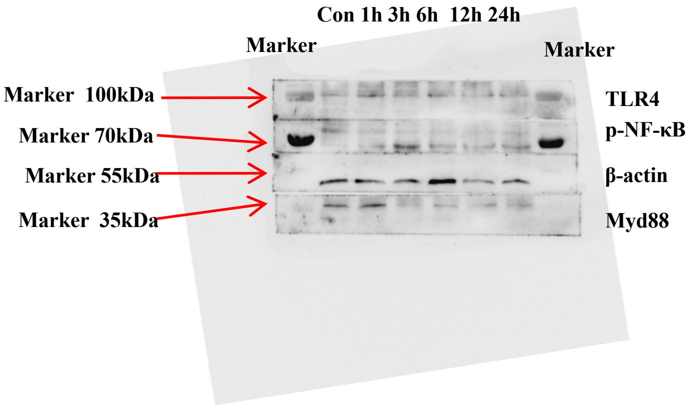

Supplementary Figure 7.

Fig.6 B

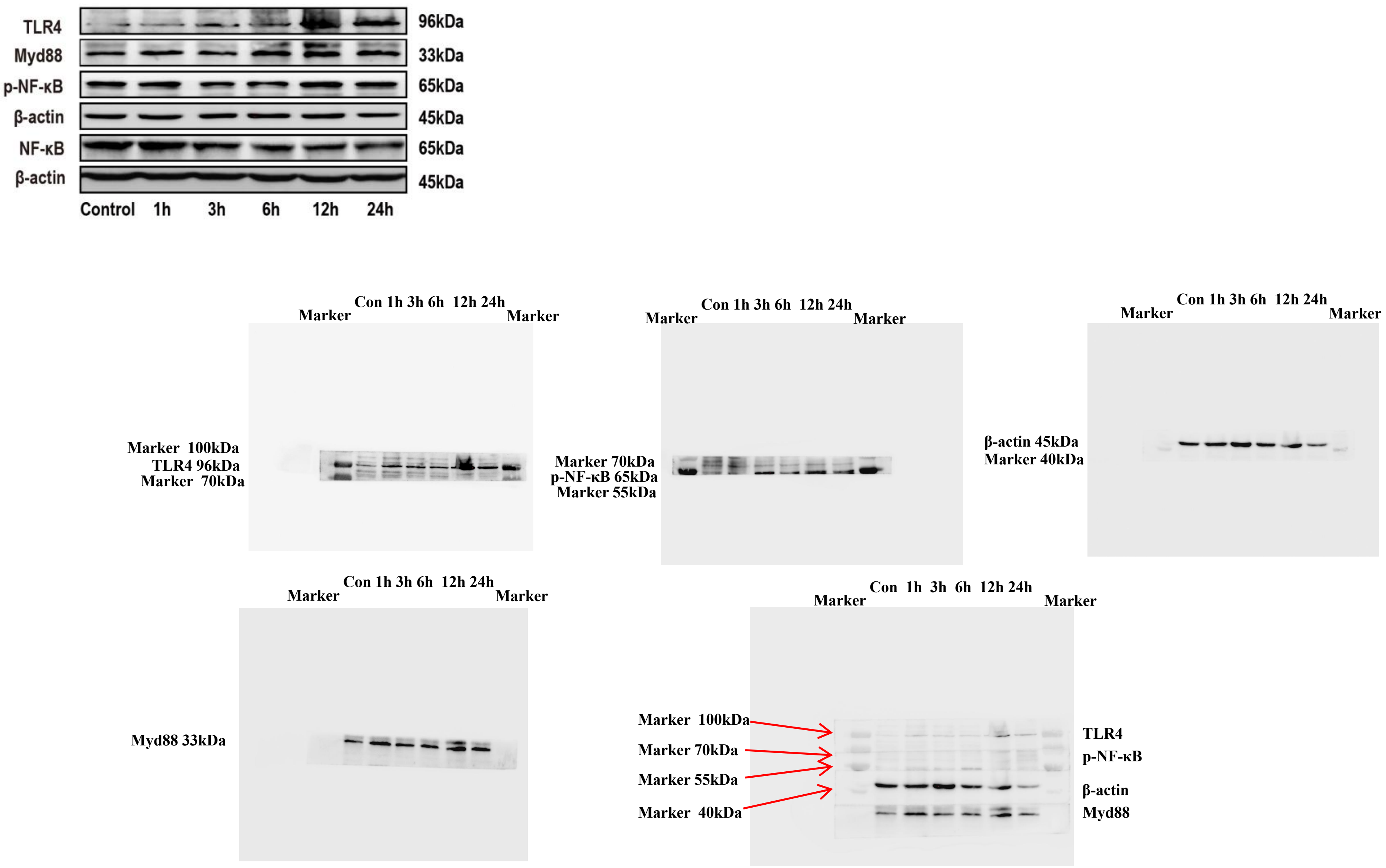

Supplementary Figure 8.

Fig.6 B

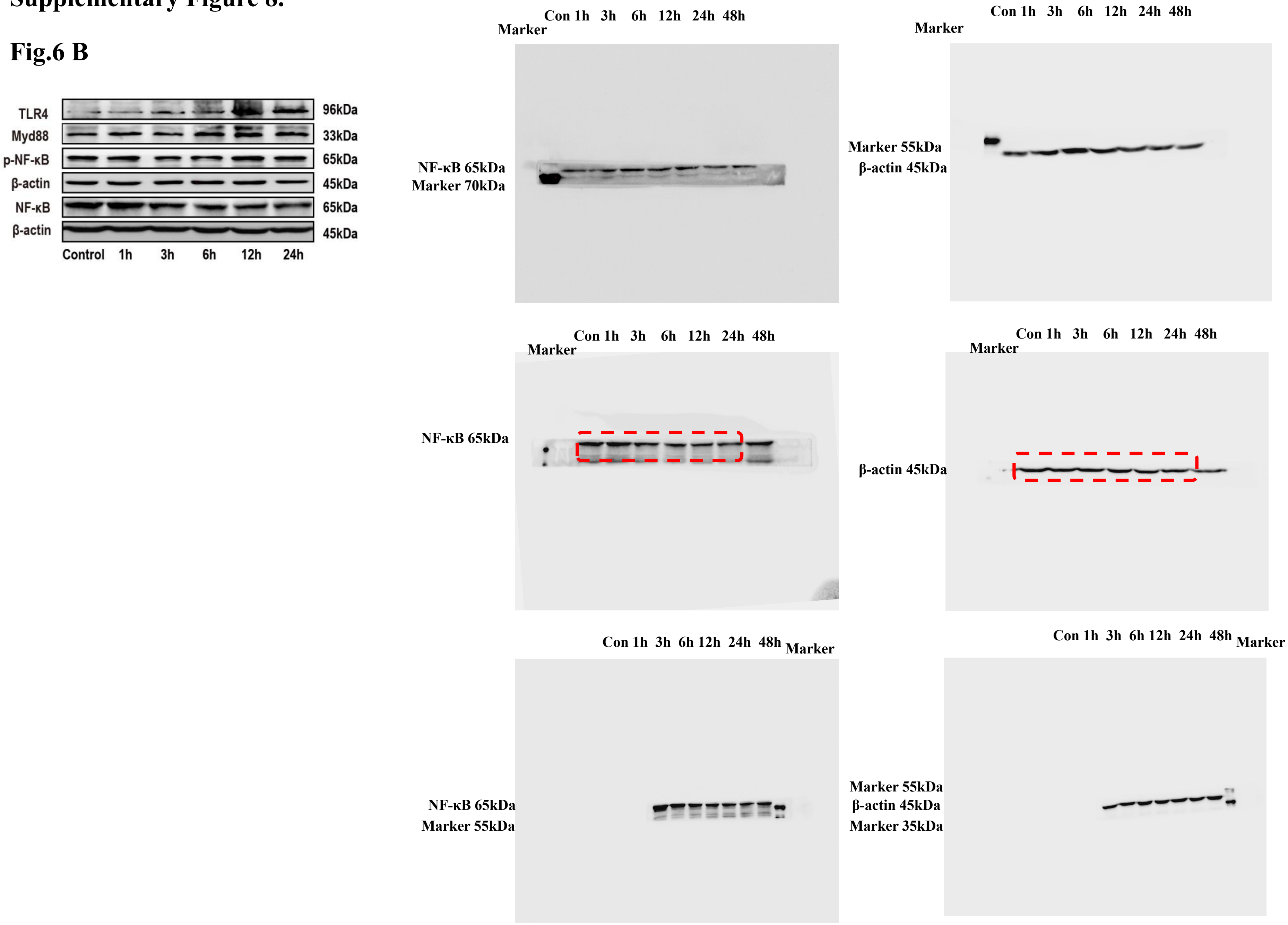

The red checkboxes are the bands presented in the manuscript image
